# IgG Glycosylation Profile and the Glycan Score Are Associated with Type 2 Diabetes in Independent Chinese Populations: A Case-Control Study

**DOI:** 10.1155/2020/5041346

**Published:** 2020-06-06

**Authors:** Zhiyuan Wu, Haibin Li, Di Liu, Lixin Tao, Jie Zhang, Baolu Liang, Xiangtong Liu, Xiaonan Wang, Xia Li, Youxin Wang, Wei Wang, Xiuhua Guo

**Affiliations:** ^1^Beijing Municipal Key Laboratory of Clinical Epidemiology, Department of Epidemiology and Health Statistics, School of Public Health, Capital Medical University, Beijing, China; ^2^Department of Mathematics and Statistics, La Trobe University, Australia; ^3^Department of Public Health, School of Medical and Health Sciences, Edith Cowan University, Perth, Australia

## Abstract

**Background:**

The relationship between the IgG glycan panel and type 2 diabetes remains unclear in Chinese population. We aimed to investigate the association of the IgG glycan profile and glycan score with type 2 diabetes.

**Methods:**

In the discovery population, 162 individuals diagnosed with type 2 diabetes and 162 matched controls from Beijing health management cohort were included. We analyzed the IgG glycan profile and composed a glycan score for type 2 diabetes. Findings were validated in the replication population from Beijing Xuanwu community cohort (280 cases and 508 controls). Area under curve (AUC) using 10-fold and bootstrap validation, net reclassification index (NRI), and integrated discrimination index (IDI) were calculated for the glycan score.

**Results:**

In the discovery population, 5 initial IgG glycans and 7 derived traits were significantly associated with type 2 diabetes after Bonferroni correction and Lasso selection, which were validated in the replication population subsequently. The glycan score composed of these IgG glycans and traits showed a strong association with type 2 diabetes (combined odds ratio (OR): 3.78) and its risk factors. In the replication population, AUC of the model involving clinical traits improved from 0.74 to above 0.90, and the values of NRI and IDI were 0.35 and 0.42, respectively, with the glycan score added.

**Conclusions:**

IgG glycosylation profiles were associated with type 2 diabetes and the glycan score may be a novel indicator for diabetes which reflected a proinflammatory status.

## 1. Background

Type 2 diabetes is a complex and chronic metabolic disease characterized by hyperglycemia and insulin resistance [1]. Type 2 diabetes has represented an extremely threatening public health issue, with a gradually increasing prevalence (projected to rise from 171 million in 2000 to 366 million in 2030) and many severe complications [2]. However, its etiological mechanism remains unclear.

Both genetic and environmental factors play a crucial role in the disease pathophysiology [3], among which glycosylation is one of the most common and substantial posttranscriptional modifications with various glycosyltransferases involved in. The *N*-oligosaccharides of glycoproteins exert important biological functions involving cellular recognition and molecular signal regulation [4]. Many proteins are modified by these glycans, and the variation of IgG glycans has been most widely described. Biantennary glycans are covalently attached at the Fc region of each heavy chain of IgG [5]. Notably, the attached glycans regulate the stability of IgG and its effector functions [6], involving antibody-dependent cell-mediated cytotoxicity (ADCC) [7] and complement-dependent cytotoxicity (CDC) [8]. The IgG glycans are emerging as potential biomarkers of various diseases, such as rheumatoid arthritis [9], ischemic stroke [10], dyslipidemia [11], kidney disease in type 1 diabetes [12], and many cancers [13–15].

Type 2 diabetes is accompanied by glucose metabolic disorder and proinflammatory status [16] while the specific IgG glycan could switch its role between pro- and anti-inflammatory functions. Meanwhile, the variation of IgG glycans has been linked to various clinical risk factors of type 2 diabetes, such as body mass index (BMI), blood pressure, and dyslipidemia [11, 17]. Recently, the inflammatory-related functions of IgG glycans in type 2 diabetes and fasting blood glucose (FBG) abnormality have been reported in European [18] and Chinese populations [19]. Although both studies have identified some type 2 diabetes-specific or FBG-specific IgG glycans, the solitary glycan presented only a relatively small and unstable association with disease status. The disease variation that the solitary glycan could explain was very limited.

We hypothesized that the IgG glycan score could integrally and robustly evaluate the status of type 2 diabetes [20]. Hence, this study is aimed at investigating the association of the IgG glycan profile and glycan score with type 2 diabetes in a matched case-control cohort, followed by validation in another independent Chinese population.

## 2. Methods

### 2.1. Study Design and Population

All 162 new cases of diabetes between Dec 2014 and Jun 2016 and 162 matched controls, from the Beijing health management cohort, were enrolled in the discovery population. The Beijing health management cohort is an ongoing population-based study of participants aged ≥18 years for metabolism-related disease research [21]. 280 cases and 508 natural controls, from the Beijing Xuanwu community cohort [19], were recruited in the replication population according to the inclusion and exclusion criteria. All the participants in this study were asked to participate in clinical measures (physical and biochemical examinations), and the fasting blood samples were also taken. Participants were required to meet the following inclusion criteria: (1) signed informed consent prior to participation, (2) at least 18 years old, and (3) enough clinical data to judge the type 2 diabetes status. Individuals were excluded based on the following criteria: (1) pregnant or lactating women, (2) history of mental illness or infectious disease, and (3) history of other types of diabetes, cardio-cerebrovascular diseases, liver disease, renal failure, cancers, or autoimmune diseases. This study was approved by the Capital Medical University Ethics Committee and conducted according to the principles of the Declaration of Helsinki. Written informed consent was obtained at the beginning of the study.

### 2.2. Measurement of Blood Glucose

The blood glucose concentrations were measured by the glucose oxidase-peroxidase method (Mind Bioengineering Co. Ltd., Shanghai, China). The FBG was defined as the glucose concentrations before breakfast after overnight fasting (no food, except drinking water, for at least 8-10 hours), while two-hour postprandial blood glucose (PBG) was measured after 2 hours from the beginning of meals. Both FBG and PBG are commonly used in clinical diabetes diagnosis, reflecting the functional reserve of islet beta cells. Type 2 diabetes was diagnosed by physicians according to the ADA and WHO criteria as follows: FBG ≥ 7.0 mmol/L, PBG ≥ 11.1 mmol/L, or regular use of antidiabetes drugs.

### 2.3. Covariates

The demographic characteristics (age and sex) of participants were collected by questionnaires. Weight and height measurements were carried out in the physical examination. The BMI was calculated by the formula weight (in kilograms)/height^2^ (in meters squared); the normal range was defined as 18.5 ≤ BMI < 24.0 (kg/m^2^) according to the WHO criteria for the Asian population. Systolic blood pressure (SBP) and diastolic blood pressure (DBP) were measured twice on the right arm using a standard mercury sphygmomanometer after the subjects had rested at least 10 min in a sitting position. High blood pressure (HBP) was defined as SBP ≥ 140 or DBP ≥ 90 according to the WHO standard. Serum total cholesterol (TC) and high-density lipoprotein cholesterol (HDL-cholesterol) were measured with an Olympus Automatic Biochemical Analyzer (Hitachi 747; Tokyo, Japan). Non-high-density lipoprotein cholesterol (nonHDL-cholesterol) was defined as the difference between TC and HDL-cholesterol.

### 2.4. IgG Glycosylation Analysis

IgG glycan analyses were conducted on participants both in the discovery and replication populations. IgG isolation, glycan release, labeling, and detection were executed as described previously [22]. Briefly, IgG protein was isolated from diluted plasma using 96-well protein G monolithic plates, washed in 1x phosphate-buffered saline (PBS), eluted with 0.1 M formic acid, and neutralized with 1 M ammonium bicarbonate. Dried IgG was denatured with 30 *μ*L sodium dodecyl sulfate (SDS) and 10 *μ*L Igepal-CA630 (4%). The glycans were released with 2 units of PNGase F in 10 *μ*L 5x PBS and incubated at 37°C for 20 h. Right after the completion of this step, released glycans were labeled with 35 *μ*L 2-AB at 65°C for 3 h and then purified, washed, and eluted using hydrophilic interaction liquid chromatography solid phase extraction. Finally, 24 IgG glycan peaks (GP) were measured by using an ultra-performance liquid chromatography platform (Waters, America); the structures of GPs were reported previously [18].

In both populations, the plasma samples were detected in the same manner into 24 peaks, and each glycan amount was expressed as the percentage of the total integrated peak area. An additional 54 derived glycan traits (IGP) describing the relative abundances of galactosylation, sialylation, bisecting *N*-acetylglucosamine (GlcNAc), core fucosylation, and mannose were calculated from the 24 directly measured GPs. IgG glycan expressions were normalized followed by log transformation and batch-effect correction.

### 2.5. Statistical Analysis

Continuous variables adhering to the normal distribution were represented as the mean ± standard deviation (SD); otherwise, the interquartile range (P_25_-P_75_) was substituted. The differences of continuous variables between the two groups were tested by the independent sample *t* tests or the Mann–Whitney tests. Categorical variables were represented as *n* (proportion), and the differences were tested by the chi-square tests. Data analysis was performed using SAS software (version 9.2). All reported *P* values were two-tailed, and *P* < 0.05 was considered statistically significant.

Propensity score matching (PSM) was used in the discovery cohort to match controls (1 : 1) for the type 2 diabetes patients. 342 subjects (162 cases and 162 controls) were recruited after age, sex, and BMI were considered in the PSM model. Logistics regression models were used to investigate the associations of the initial IgG glycans and derived traits with type 2 diabetes. Bonferroni correction was applied for 78 tests, and *P* values < 6.41*E*-4 were considered statistically significant. The IgG glycans and derived traits both selected by logistics model and lasso model were used to compose the glycan score with coefficients set by lasso regression model. The formula of this glycan score is as follows: Score = −0.124∗GP3 − 0.428∗GP5 − 0.093∗GP20 + 0.249∗GP22 + 0.110∗GP24 + 0.520∗IGP32 + 0.334∗IGP42 + 0.152∗IGP45 − 0.584∗IGP46 − 0.316∗IGP47 + 0.380∗IGP60 − 0.125∗IGP69.

Subsequently, the results of the primary analyses were validated in an independent replication population. All the analyses presented above were performed using R (version 3.3.2) packages: MatchIt and glmnet.

In addition, the discrimination capacity of the glycan score was evaluated in the replication population. Three models were considered: model 1, involving the clinical traits (age, sex, BMI, HBP, HDL-cholesterol, nonHDL-cholesterol); model 2, involving the glycan score; and model 3, involving the combination of the clinical traits and the glycan score. For prediction analyses (to infer an outcome given the covariates in the statistical sense), we fitted the logistic models with 10-fold cross-validation and bootstrap strategy. In 10-fold cross-validation, the whole samples were randomly divided into 10 subgroups, where one subgroup served as the testing set and the other 9 subgroups served as the training set. This process was repeated for all folds, and the average value of area under curve (AUC) was calculated. In bootstrap validation, we obtained distinct data sets by repeatedly sampling observations from the original data set, rather than repeatedly obtaining independent data sets from the population, thus to provide an estimate of the accuracy and quantify the uncertainty of the logistic models [23]. We also computed the value of net reclassification index (NRI) and integrated discrimination index (IDI) to compare the models with and without the glycan score. NRI focused on reclassification tables constructed separately for subjects with and without events and quantified the correct movement in classification for models with and without the new marker, while IDI quantified jointly the overall improvement in sensitivity and specificity over all possible cut-offs [24]. All the analyses presented above were performed using the R packages: pROC, fproc, cvAUC, and predictABEL.

## 3. Results

### 3.1. Participant Characteristics

In the discovery population, 162 cases with type 2 diabetes and 162 matched controls were included. The controls were selected according to age, sex, and BMI. In the replication population, 280 cases with type 2 diabetes and 508 natural controls were recruited. The characteristics of the subjects in the discovery population and replication population are presented in Table 1.

### 3.2. Associations of the IgG Glycan Score with Type 2 Diabetes

**Figure 1:**
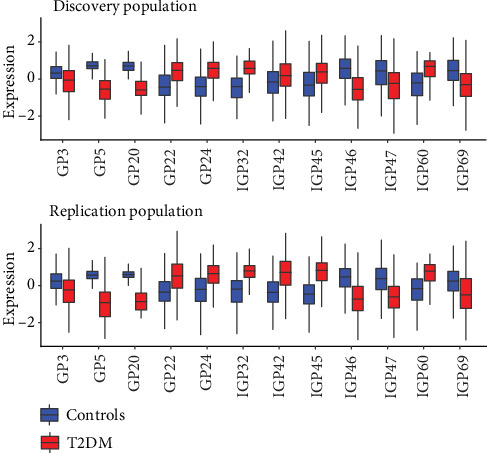
Distribution of IgG glycan peaks and traits associated with type 2 diabetes. Upper half: distribution of glycans in the discovery population. Bottom half: distribution of glycans in the replication population. This figure shows the relative expression of each glycan for controls (blue) and cases (red).

Detailed IgG glycan structures were reported previously [25], and the characteristics of each structure was explained in Supplementary Table S1.  Table 2 showed that the 12 IgG glycans were significantly associated with type 2 diabetes in the discovery population which were subsequently validated in the replication population. The boxplots of these 12 IgG glycans in the discovery population and replication population are presented in Figure 1. After that, the 12 glycans were used to compose the glycan score, among which 6 glycans were increased and 6 were decreased in the type 2 diabetes cases. The OR and *P* values were combined with meta-analysis using the weighted *z*-transform method. A higher glycan score was associated with a stronger probability of type 2 diabetes, and the combined OR value was 3.78 (95% CI: 3.07-4.49). The coefficients and *P* values of all the 78 IgG glycans were shown in Supplementary Table S2.

**Figure 2:**
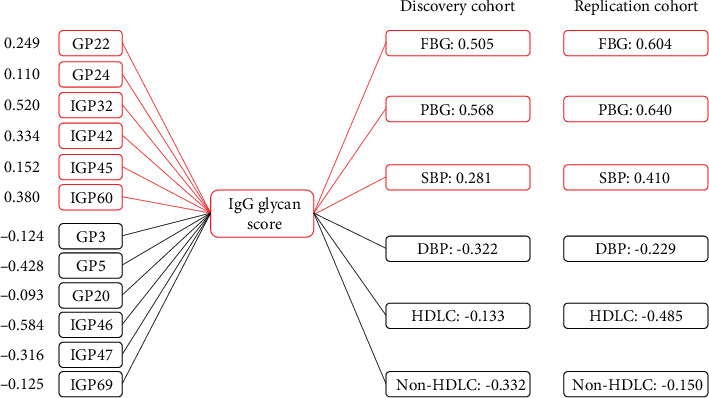
Composition of the IgG glycan score and its correlations with clinical traits in the discovery and replication populations. Red: positively correlated with the glycan score; blue: negatively correlated with the glycan score; FBG: fasting blood glucose; PBG: two-hour postprandial blood glucose; SBP: systolic blood pressure; DBP: diastolic blood pressure; HDL-cholesterol: high-density lipoprotein cholesterol; nonHDL-cholesterol: non-high-density lipoprotein cholesterol.

Figure 2 illustrated the contribution of each IgG glycan to the glycan score and the correlation with clinical traits which were also the risk factors of type 2 diabetes. Both in the discovery and replication populations, the glycan score presented significant univariate associations with all these clinical traits consistently (all *P* values < 0.001), while positively correlated with SBP, FBG, and PBG and negatively correlated with DBP, HDL-cholesterol, and nonHDL-cholesterol.

### 3.3. Discrimination Capacity of the IgG Glycan Score for Type 2 Diabetes

The discrimination capacity of the glycan score was evaluated in the replication population, and the AUC values for the clinical traits, the glycan score, and their combination are shown in Table 3. Adding the glycan score to the model containing the clinical traits could significantly improve the discrimination capacity (bootstrap: 0.742 vs. 0.918; 10-fold cross-validation: 0.744 vs. 0.923) while the NRI and IDI were 0.350 (95% CI: 0.241-0.458, *P* < 0.001) and 0.421 (95% CI: 0.398-0.493, *P* < 0.001), respectively. There was a statistically significant difference between the AUC values of the clinical variables with and without the glycan score (*P* < 0.001). However, the AUC values of the glycan score with and without the clinical variables were similar (*P* = 0.08) which implied the glycan score could reflect the clinical characteristics to some extent.

## 4. Discussion

In this study, we described the association of the IgG glycan profile and glycan score with type 2 diabetes in Chinese population. We found and replicated that GP3, GP5, GP20, GP22, GP24, and several IgG-derived traits could compose a glycan score to discriminate the type 2 diabetes individuals from the health controls effectively for the first time. Additionally, the glycan score was also correlated with some clinical traits, reflecting the influence of these clinical factors partly. Notably, the AUC of the IgG glycan score along for type 2 diabetes was above 0.90 using10-fold and bootstrap validation.

Type 2 diabetes is a polygenic and multifactorial disease in which genetic and environmental factors interact [16, 26] while the IgG glycans could reflect both the genetic and posttranscriptional modifications [27–29]. The changes of IgG glycans have been reported to be associated with various diseases, involving rheumatoid arthritis, cancers, and many chronic metabolic diseases [28]. In this study, we found that GP3, GP5, and GP20 were increased in the type 2 diabetes individuals while GP22 and GP24 were decreased. The changes of directly measured IgG glycans were in accordance with an increase of structures with bisecting GlcNac, a high percentage of disialylation, and a decrease of simple glycan structures. Meanwhile, the derived traits associated with type 2 diabetes reflected an increase of complex structures (biantennary glycan structures in total neutral IgG glycans and disialylation of fucosylated digalactosylated structures with bisecting GlcNAc), an increase of high mannose structures, a decrease of monogalactosylation structures, and a low percentage of fucosylation in digalactosylated structures with and without bisecting GlcNAc.

The results were largely in line with previous studies of IgG glycans and total serum/plasma protein glycomics profile in type 2 diabetes or its risk factors [10–12, 17, 30–33]. Previous studies have shown that complex glycan structures (IGP32 and IGP42) were excessively expressed in response to some inflammatory diseases, such as ulcerative colitis [34] and type 1 diabetes [30]. Individuals with type 2 diabetes also suffered from the chronic inflammation, and IgG proteins were sensitive to physical inflammatory stress. Therefore, the evaluated proportion of multibranched and complex glycan structures may be induced by the chronic inflammation. Additionally, the presence of bisecting GlcNAc [35] and lack of core fucosylation [36] were thought to strengthen the ADCC effect of IgG, while the decreased percentage of galactosylation (accompanied by lowered percentage of disialylation) could magnify the CDC effect, thus strengthening its proinflammatory function [37, 38]. These changes indicated the glycan score could represent a proinflammatory signal in type 2 diabetes. Similarly, Lemmers et al. reported the IgG glycan patterns associated with type 2 diabetes based on a European population and found a decrease of galactosylation and sialylation structures, a decrease of fucosylated structures without bisecting GlcNAc, and an increase of fucosylated structures with bisecting GlcNac [18]. In our study, decreased galactosylation (GP8n) was also observed. However, the proportions of fucosylated structures with (GP7n, FA2BG2S1) and without (FA2FG2S1) bisecting GlcNAc both decreased. Therefore, the role of fucosylation in structures with and without bisecting GlcNAc for type 2 diabetes warrants further investigation. In addition, we found an increased level of high mannose glycan structures in total neutral IgG glycans (GP6n) which was not previously reported in type 2 diabetes. High mannose of IgG glycans were reported to enhance the ADCC effect and exert a proinflammatory function [39, 40]. The role of IgG glycans with high mannose in type 2 diabetes needs to be further studied.

The strength of our study was that we explored the IgG glycan profile of type 2 diabetes in Chinese population and we composed and validated the glycan score to discriminate the type 2 diabetes individuals from healthy controls. The glycan score could comprehensively reflect the IgG glycan changes of type 2 diabetes than solidary glycan. Additionally, the glycan score was strongly associated with type 2 diabetes with a combined OR of 3.78. Meanwhile, the glycan score was correlated with several clinical traits which were also the risk factors of type 2 diabetes, and it could reflect more information than these clinical traits. The AUC of model involving clinical traits improved from 0.74 to 0.90 when the glycan score added. However, the results should be interpreted in the context of some limitations. First, the case-control design could lead to an overestimation of the AUC of the ROC curve, and we could not claim an casual correlation. Also, due to the lack of prospective follow-up, we could not exclude that several individuals of the control population would develop type 2 diabetes by the time they reach the age of the cases, as the controls were substantially younger than the cases in the replication population. Second, we failed to collect the medication information of the cases, and the antidiabetics medication could affect glucose level, thus having a potential effect on the glycosylation pattern. Third, our study focused on the Chinese population, and more collaborations were needed to create a larger sample size and ensure population representation.

## 5. Conclusions

The IgG glycan score was associated with type 2 diabetes that reflected a proinflammatory status. These findings implied that the glycan score may be a potential and comprehensive indicator for type 2 diabetes and complex inflammatory status which warrants further investigation.

## Figures and Tables

**Table 1:** The characteristics of participants in the discovery and replication populations.

	Overall	Controls	Type 2 diabetes	*P* value
Discovery population	*n* = 324	*n* = 162	*n* = 162	
Age (years)^a^	51.40 (±7.65)	51.39 (±7.35)	51.41 (±7.97)	0.997
Sex (male/female)^b^	72/252	36/126	36/126	1.000
BMI (<18.5/18.5~23.9/>23.9)^b^	1/69/254	0/35/127	1/34/127	0.602
HBP (yes/no)^b^	101/223	55/107	46/116	0.337
HDL-cholesterol (mmol/L)^c^	1.13 (1.10-1.57)	1.45 (1.25-1.65)	1.19 (1.04-1.37)	<0.001
nonHDL-cholesterol (mmol/L)^c^	3.50 (2.92-4.10)	3.62 (3.06-4.15)	3.29 (2.75-3.95)	0.007
Replication population	*n* = 788	*n* = 508	*n* = 280	
Age (years)^a^	49.92 (±7.75)	42.15 (±9.77)	64.00 (±6.73)	<0.001
Sex (male/female)^b^	338/450	91/417	247/33	<0.001
BMI (<18.5/18.5 ~ 23.9/>23.9)^b^	26/318/444	26/277/205	0/41/239	<0.001
HBP (yes/no)^b^	144/644	58/450	86/194	<0.001
HDL-cholesterol (mmol/L)^c^	1.53 (1.25-1.79)	1.68 (1.47-1.86)	1.21 (1.04-1.44)	<0.001
nonHDL-cholesterol (mmol/L)^c^	3.22 (2.64-3.76)	3.30 (2.84-3.83)	2.98 (2.29-3.58)	<0.001

^a^Mean (±SD) is given; ^b^numbers of each category are given; ^c^median (P_25_-P_75_) is given.

**Table 2:** Associations of the IgG glycans and glycan score for type 2 diabetes in the discovery and replication populations and meta-analyses.

Glycan peak	Glycan structure^a^	Discovery population (*n* = 324)		Replication population (*n* = 788)		Meta-analyses	
		OR	*P* value^b^	OR	*P* value^b^	OR	*P* value^b^
Upregulated							
GP22	A2BG2S2	2.520	<0.0001	2.535	<0.0001	2.531	<0.0001
GP24	FA2BG2S2	3.611	<0.0001	3.533	<0.0001	3.556	<0.0001
IGP32	FBG2S2/(FBG2+FBG2S1+FBG2S2)	5.392	<0.0001	8.717	<0.0001	6.621	<0.0001
IGP42	GP2n	1.542	0.0003	3.826	<0.0001	1.913	<0.0001
IGP45	GP6n	1.793	<0.0001	5.668	<0.0001	2.132	<0.0001
IGP60	FG1n total/G1n	4.038	<0.0001	4.551	<0.0001	4.349	<0.0001
Downregulated							<0.0001
GP3	A2B	0.392	<0.0001	0.351	<0.0001	0.361	<0.0001
GP5	M5	0.106	<0.0001	0.021	<0.0001	0.026	<0.0001
GP20	FA2FG2S1	0.158	<0.0001	0.017	<0.0001	0.020	<0.0001
IGP46	GP7n	0.232	<0.0001	0.183	<0.0001	0.195	<0.0001
IGP47	GP8n	0.510	<0.0001	0.272	<0.0001	0.313	<0.0001
IGP69	FBG2n/G2n	0.350	<0.0001	0.486	<0.0001	0.435	<0.0001
Glycan score	—	3.309	<0.0001	7.145	<0.0001	3.782	<0.0001

^a^Structure abbreviations: F: core fucose; A: number of antennas; B: bisecting GlcNAc; M: number of mannose residues; Gx: number of galactoses; Sx: number of sialic acids linked to galactose; n: neutral glycans. ^b^*P* values < 6.41*E*-4 was considered statistically significant.

**Table 3:** AUCs of discrimination models calculated using bootstrap validation and 10-fold cross-validation in the replication population.

	Bootstrap (*n* = 1000)			Cross-validation (10-fold)		
	AUC	Std error	95% CI	AUC	Std error	95% CI
Model 1	0.742	0.023	0.683-0.828	0.744	0.024	0.690-0.811
Model 2	0.902	0.018	0.881-0.943	0.916	0.018	0.883-0.949
Model 3	0.918	0.015	0.890-0.949	0.923	0.014	0.900-0.954

Model 1: clinical traits (age, sex, BMI, HBP, HLDL-cholesterol, nonHDL-cholesterol) included; model 2: glycan score included; model 3: clinical traits and glycan score included.

## Data Availability

The data used to support the findings of this study are available from the corresponding author upon request.

## Abbreviations

AUC: Area under curve
NRI: Net reclassification index
IDI: Integrated discrimination index
OR: Odds ratio
ADCC: Antibody-dependent cell-mediated cytotoxicity
CDC: Complement-dependent cytotoxicity
BMI: Body mass index
FBG: Fasting blood glucose
PBG: Two-hour postprandial blood glucose
SBP: Systolic blood pressure
DBP: Diastolic blood pressure
TC: Total cholesterol
HDL-cholesterol: High-density lipoprotein cholesterol
nonHDL-cholesterol: Non-high-density lipoprotein cholesterol
PBS: Phosphate-buffered saline
SDS: Sodium dodecyl sulfate
PSM: Propensity score matching.

## Supplementary Materials

Supplementary Materials Supplementary Table S1: the structures and descriptions of the IgG glycans. Supplementary Table S2: the associations of all the 78 IgG glycans with type 2 diabetes for the discovery and replication populations.

## References

[1] Stumvoll M., Goldstein B. J., van Haeften T. W. (2005). Type 2 diabetes: principles of pathogenesis and therapy. *Lancet*.

[2] Wild S., Roglic G., Green A., Sicree R., King H. (2004). Global prevalence of diabetes: estimates for the year 2000 and projections for 2030. *Diabetes Care*.

[3] the DIAbetes Genetics Replication And Meta-analysis (DIAGRAM) Consortium (2012). Large-scale association analysis provides insights into the genetic architecture and pathophysiology of type 2 diabetes. *Nature Genetics*.

[4] Skropeta D. (2009). The effect of individual N-glycans on enzyme activity. *Bioorganic & Medicinal Chemistry*.

[5] Harada H., Kamei M., Tokumoto Y. (1987). Systematic fractionation of oligosaccharides of human immunoglobulin G by serial affinity chromatography on immobilized lectin columns. *Analytical Biochemistry*.

[6] Kobata A. (2008). The N-linked sugar chains of human immunoglobulin G: their unique pattern, and their functional roles. *Biochimica et Biophysica Acta*.

[7] Masuda K., Kubota T., Kaneko E. (2007). Enhanced binding affinity for FcgammaRIIIa of fucose-negative antibody is sufficient to induce maximal antibody-dependent cellular cytotoxicity. *Molecular Immunology*.

[8] Bohm S., Schwab I., Lux A., Nimmerjahn F. (2012). The role of sialic acid as a modulator of the anti-inflammatory activity of IgG. *Seminars in Immunopathology*.

[9] Parekh R. B., Dwek R. A., Sutton B. J. (1985). Association of rheumatoid arthritis and primary osteoarthritis with changes in the glycosylation pattern of total serum IgG. *Nature*.

[10] Liu D., Zhao Z., Wang A. (2018). Ischemic stroke is associated with the pro-inflammatory potential of N-glycosylated immunoglobulin G. *Journal of Neuroinflammation*.

[11] Liu D., Chu X., Wang H. (2018). The changes of immunoglobulin G N-glycosylation in blood lipids and dyslipidaemia. *Journal of Translational Medicine*.

[12] Bermingham M. L., Colombo M., McGurnaghan S. J. (2017). N-glycan profile and kidney disease in type 1 diabetes. *Diabetes Care*.

[13] Yi C. H., Weng H. L., Zhou F. G. (2015). Elevated core-fucosylated IgG is a new marker for hepatitis B virus-related hepatocellular carcinoma. *Oncoimmunology*.

[14] Kanoh Y., Mashiko T., Danbara M. (2004). Changes in serum IgG oligosaccharide chains with prostate cancer progression. *Anticancer Research*.

[15] Ruhaak L. R., Barkauskas D. A., Torres J. (2015). The serum immunoglobulin G glycosylation signature of gastric cancer. *EuPA open Proteomics*.

[16] Wang X., Bao W., Liu J. (2012). Inflammatory markers and risk of type 2 diabetes: a systematic review and meta-analysis. *Diabetes Care*.

[17] Nikolac Perkovic M., Pucic Bakovic M., Kristic J. (2014). The association between galactosylation of immunoglobulin G and body mass index. *Progress in Neuro-Psychopharmacology and Biological Psychiatry*.

[18] Lemmers R. F. H., Vilaj M., Urda D. (2017). IgG glycan patterns are associated with type 2 diabetes in independent European populations. *Biochimica et Biophysica Acta (BBA) - General Subjects*.

[19] Ge S., Wang Y., Song M. (2018). Type 2 diabetes mellitus: integrative analysis of multiomics data for biomarker discovery. *OMICS: A Journal of Integrative Biology*.

[20] Menni C., Gudelj I., Macdonald-Dunlop E. (2018). Glycosylation profile of immunoglobulin G is cross-sectionally associated with cardiovascular disease risk score and subclinical atherosclerosis in two independent cohorts. *Circulation Research*.

[21] Liu J., Zhao Z., Mu Y. (2018). Gender differences in the association between serum uric acid and prediabetes: a six-year longitudinal cohort study. *International Journal of Environmental Research and Public Health*.

[22] Trbojevic-Akmacic I., Vilaj M., Lauc G. (2016). High-throughput analysis of immunoglobulin G glycosylation. *Expert Review of Proteomics*.

[23] Carpenter J., Bithell J. (2000). Bootstrap confidence intervals: when, which, what? A practical guide for medical statisticians. *Statistics in Medicine*.

[24] Pencina M. J., D' Agostino R. B., D' Agostino R. B., Vasan R. S. (2008). Evaluating the added predictive ability of a new marker: from area under the ROC curve to reclassification and beyond. *Statistics in Medicine*.

[25] Pučić M., Knežević A., Vidič J. (2011). High throughput isolation and glycosylation analysis of IgG-variability and heritability of the IgG glycome in three isolated human populations. *Molecular & Cellular Proteomics*.

[26] Ding D., Chong S., Jalaludin B., Comino E., Bauman A. E. (2015). Risk factors of incident type 2-diabetes mellitus over a 3-year follow-up: results from a large Australian sample. *Diabetes Research and Clinical Practice*.

[27] Wahl A., van den Akker E., Klaric L. (2018). Genome-wide association study on immunoglobulin G glycosylation patterns. *Frontiers in Immunology*.

[28] Liu D., Li Q., Zhang X. (2019). Systematic review: immunoglobulin GN-glycans as next-generation diagnostic biomarkers for common chronic diseases. *Omics : a Journal of Integrative Biology*.

[29] Mimura Y., Katoh T., Saldova R. (2018). Glycosylation engineering of therapeutic IgG antibodies: challenges for the safety, functionality and efficacy. *Protein and Cell*.

[30] Testa R., Vanhooren V., Bonfigli A. R. (2015). N-glycomic changes in serum proteins in type 2 diabetes mellitus correlate with complications and with metabolic syndrome parameters. *PLoS One*.

[31] Wang Y., Klarić L., Yu X. (2016). The association between glycosylation of immunoglobulin G and hypertension: a multiple ethnic cross-sectional study. *Medicine*.

[32] Krištić J., Vučković F., Menni C. (2014). Glycans are a novel biomarker of chronological and biological ages. *The Journals of Gerontology: Series A*.

[33] Knežević A., Gornik O., Polašek O. (2010). Effects of aging, body mass index, plasma lipid profiles, and smoking on human plasma N-glycans. *Glycobiology*.

[34] Miyahara K., Nouso K., Saito S. (2013). Serum glycan markers for evaluation of disease activity and prediction of clinical course in patients with ulcerative colitis. *PLoS One*.

[35] Ruhaak L. R., Uh H. W., Beekman M. (2010). Decreased levels of bisecting GlcNAc glycoforms of IgG are associated with human longevity. *PLoS One*.

[36] Shields R. L., Lai J., Keck R. (2002). Lack of fucose on human IgG1 N-linked oligosaccharide improves binding to human Fcgamma RIII and antibody-dependent cellular toxicity. *The Journal of Biological Chemistry*.

[37] Anthony R. M., Ravetch J. V. (2010). A novel role for the IgG Fc glycan: the anti-inflammatory activity of sialylated IgG Fcs. *Journal of Clinical Immunology*.

[38] Kaneko Y., Nimmerjahn F., Ravetch J. V. (2006). Anti-inflammatory activity of immunoglobulin G resulting from Fc sialylation. *Science*.

[39] Zhou Q., Shankara S., Roy A. (2008). Development of a simple and rapid method for producing non-fucosylated oligomannose containing antibodies with increased effector function. *Biotechnology and Bioengineering*.

[40] Goetze A. M., Liu Y. D., Zhang Z. (2011). High-mannose glycans on the Fc region of therapeutic IgG antibodies increase serum clearance in humans. *Glycobiology*.

