# Supplementary material for: IgG Glycosylation Profile and the Glycan Score Are Associated with Type 2 Diabetes in Independent Chinese Populations: A Case-Control Study
Source: J Diabetes Res. 2020 Jun 6;2020:5041346. doi: 10.1155/2020/5041346 (PMC7301241; doi:10.1155/2020/5041346)
Supplement: Supplementary Materials — Supplementary Table S1: the structures and descriptions of the IgG glycans. Supplementary Table S2: the associations of all the 78 IgG glycans with type 2 diabetes for the discovery and replication populations. [file 5041346.f1.pdf]

Supplementary Table S1: The structures and descriptions of the IgG glycans.

Structure abbreviations: F, core fucose; A, number of antenna's; B, bisecting GlcNAc; M, number of mannose residues; Gx, number of galactoses; Sx, number of sialic acids linked to galactose; n, neutral glycans.

	Structure	Description
**Glycan peak**		
GP1	FA1	monoantennary glycan with core fucose
GP2	A2	biantennary glycan
GP3	-	structure not determined
GP4	FA2	monoantennary glycan with core fucose
GP5	M5	high mannose glycan
GP6	FA2B	biantennary glycan with bisecting GlcNAc and core fucose
GP7	A2G1	monogalactosylated biantennary glycan
GP8	FA2[6]G1	monogalactosylated biantennary glycan with 1-6 linkages with core fucose
GP9	FA2[3]G1	monogalactosylated biantennary glycan with 1-3 linkages with core fucose
GP10	FA2[6]BG1	monogalactosylated biantennary glycan with 1-6 linkages with bisecting GlcNAc and core fucose
GP11	FA2[3]BG1	monogalactosylated biantennary glycan with 1-3 linkages with bisecting GlcNAc and core fucose
GP12	A2G2	digalactosylated biantennary glycan
GP13	A2BG2	digalactosylated biantennary glycan with bisecting GlcNAc
GP14	FA2G2	digalactosylated biantennary glycan with core fucose
GP15	FA2BG2	digalactosylated biantennary glycan with bisecting GlcNAc and core fucose
GP16	FA2G1S1	monogalactosylated monosialylated biantennary glycan with core fucose
GP17	A2G2S1	digalactosylated monosialylated biantennary glycan
GP18	FA2G2S1	digalactosylated monosialylated biantennary glycan with core fucose
GP19	FA2BG2S1	digalactosylated monosialylated biantennary glycan with bisecting GlcNAc and core fucose
GP20	FA2FG2S1	digalactosylated monosialylated biantennary with core and antennary fucose
GP21	A2G2S2	digalactosylated disialylated biantennary glycan

GP22	A2BG2S2	digalactosylated disialylated biantennary glycan with bisecting GlcNAc
GP23	FA2G2S2	digalactosylated disialylated biantennary glycan with core fucose
GP24	FA2BG2S2	digalactosylated disialylated biantennary glycan with bisecting GlcNAc and core fucose
**Derived trait**		
IGP24	FGS/(FG+FGS)	sialylation of fucosylated galactosylated structures without bisecting GlcNAc
IGP25	FBGS/(FBG+FBGS)	sialylation of fucosylated galactosylated structures with bisecting GlcNAc
IGP26	FGS/(F+FG+FGS)	sialylation of all fucosylated structures without bisecting GlcNAc
IGP27	FBGS/(FB+FBG+FBGS)	sialylation of all fucosylated structures with bisecting GlcNAc
IGP28	FG1S1/(FG1+FG1S1)	Monosialylation of fucosylated monogalactosylated structures
IGP29	FG2S1/(FG2+FG2S1+FG2S2)	monosialylation of fucosylated digalactosylated structures
IGP30	FG2S2/(FG2+FG2S1+FG2S2)	disialylation of fucosylated digalactosylated structures
IGP31	FBG2S1/(FBG2+FBG2S1+FBG2S2)	monosialylation of fucosylated digalactosylated structures with bisecting GlcNAc
IGP32	FBG2S2/(FBG2+FBG2S1+FBG2S2)	disialylation of fucosylated digalactosylated structures with bisecting GlcNAc
IGP33	FtotalS1/FtotalS2	ratio of all fucosylated (+/- bisecting GlyNAc) monosyalilated and disialylated structures
IGP34	FS1/FS2	ratio of fucosylated (without bisecting GlcNAc) monosialylated and disialylated structures
IGP35	FBS1/FBS2	ratio of fucosylated (with bisecting GlcNAc) monosialylated and disialylated structures
IGP36	FBStotal/FStotal	ratio of all fucosylated sialylated structures with and without bisecting GlcNAc
IGP37	FBS1/FS1	ratio of fucosylated monosialylated structures with and without bisecting GlcNAc
IGP38	FBS1/(FS1+FBS1)	the incidence of bisecting GlcNAc in all fucosylated monosialylated structures
IGP39	FBS2/FS2	ratio of fucosylated disialylated structures with and without bisecting GlcNAc
IGP40	FBS2/(FS2+FBS2)	The incidence of bisecting GlcNAc in all fucosylated disialylated structures
IGP41	GP1n	monoantennary glycan with core fucose structures in total neutral IgG glycans

IGP42	GP2n	biantennary glycan structures in total neutral IgG glycans
IGP43	GP4n	structure not determined
IGP44	GP5n	biantennary glycan with core fucose structures in total neutral IgG glycans
IGP45	GP6n	high mannose glycan structures in total neutral IgG glycans
IGP46	GP7n	biantennary glycan with bisecting GlcNAc and core fucose structures in total neutral IgG glycans
IGP47	GP8n	monogalactosylated biantennary glycan structures in total neutral IgG glycans
IGP48	GP9n	monogalactosylated biantennary glycan with 1-6 linkages structures in total neutral IgG glycans
IGP49	GP10n	monogalactosylated biantennary glycan with 1-3 linkages structures in total neutral IgG glycans
IGP50	GP11n	monogalactosylated biantennary glycan with 1-6 linkages with bisecting GlcNAc structures in total neutral IgG glycans
IGP51	GP12n	monogalactosylated biantennary glycan with 1-3 linkages with bisecting GlcNAc structures in total neutral IgG glycans
IGP52	GP13n	digalactosylated biantennary glycan structures in total neutral IgG glycans
IGP53	GP14n	digalactosylated biantennary glycan with bisecting GlcNAc structures in total neutral IgG glycans
IGP54	GP15n	digalactosylated biantennary glycan with core fucose structures in total neutral IgG glycans
IGP55	G0n	agalactosylated structures in total neutral IgG glycans
IGP56	G1n	monogalactosylated structures in total neutral IgG glycans
IGP57	G2n	digalactosylated structures in total neutral IgG glycans
IGP58	Fn total	all fucosylated (+/- bisecting GlcNAc) structures in total neutral IgG glycans
IGP59	FG0n total/G0n	fucosylation of agalactosylated structures
IGP60	FG1n total/G1n	fucosylation of monogalactosylated structures
IGP61	FG2n total /G2n	fucosylation of digalactosylated structures
IGP62	Fn	fucosylated (without bisecting GlcNAc) structures in total neutral IgG glycans
IGP63	FG0n/G0n	fucosylation (without bisecting GlcNAc) of agalactosylated structures
IGP64	FG1n/G1n	fucosylation (without bisecting GlcNAc) of

		monogalactosylated structures
IGP65	FG2n/G2n	fucosylation (without bisecting GlcNAc) of digalactosylated structures
IGP66	FBn	fucosylated (with bisecting GlcNAc) structures in total neutral IgG glycans
IGP67	FBG0n/G0n	fucosylation (with bisecting GlcNAc) of agalactosylated structures
IGP68	FBG1n/G1n	fucosylation (with bisecting GlcNAc) of monogalactosylated structures
IGP69	FBG2n/G2n	fucosylation (with bisecting GlcNAc) of digalactosylated structures
IGP70	FBn/Fn	ratio of fucosylated structures with and without bisecting GlcNAc
IGP71	FBn/Fn total	the incidence of bisecting GlcNAc in all fucosylated structures in total neutral IgG glycans
IGP72	Fn/(Bn + FBn)	ratio of fucosylated non-bisecting GlcNAc structures and all structures with bisecting GlcNAc
IGP73	Bn/(Fn + FBn)	ratio of structures with bisecting GlcNAc and all fucosylated structures (+/- bisecting GlcNAc)
IGP74	FBG2n/FG2n	ratio of fucosylated digalactosylated structures with and without bisecting GlcNAc
IGP75	FBG2n /(FG2n + FBG2n)	the incidence of bisecting GlcNAc in all fucosylated digalactosylated structures in total neutral IgG glycans
IGP76	FG2n/(BG2n + FBG2n)	ratio of fucosylated digalactosylated non-bisecting GlcNAc structures and all digalactosylated structures with bisecting GlcNAc
IGP77	BG2n/(FG2n + FBG2n)	ratio of digalactosylated structures with bisecting GlcNAc and all fucosylated digalactosylated structures (+/- bisecting GlcNAc)

Supplementary Table S2: The associations of all the 78 IgG glycans with type 2 diabetes for the discovery and replication populations.

Each of the IgG glycan was explored in the univariate logistics models and *P*-values<6.41E-4 was considered statistically significant.

	**Discovery population**				**Replication population**			
	β	Std err	*P* value	OR	β	Std err	*P* value	OR
GP1	0.2445	0.1172	0.037	1.277	0.6449	0.0886	<.0001	1.906
GP10	-0.64	0.1291	<.0001	0.527	-0.2179	0.0754	0.0039	0.804
GP11	-0.7474	0.1339	<.0001	0.474	0.0263	0.0745	0.724	1.027
GP12	-0.3036	0.1146	0.0081	0.738	-0.5796	0.0807	<.0001	0.56
GP13	-1.5199	0.1861	<.0001	0.219	-2.9699	0.2054	<.0001	0.051
GP14	-0.35	0.1177	0.0029	0.705	-1.2409	0.1065	<.0001	0.289
GP15	-1.2732	0.1648	<.0001	0.28	-1.8241	0.1347	<.0001	0.161
GP16	-0.1921	0.1131	0.0895	0.825	-0.1346	0.0745	0.0707	0.874
GP17	-0.2942	0.1147	0.0103	0.745	-0.8115	0.099	<.0001	0.444
GP18	-0.4567	0.2021	0.0238	0.633	-1.4956	0.1411	<.0001	0.224
GP19	0.4404	0.1411	0.0018	1.553	0.7382	0.0956	<.0001	2.092
GP2	0.426	0.1206	0.0004	1.531	1.3758	0.1126	<.0001	3.958
GP20	-1.8423	0.2091	<.0001	0.158	-4.0981	0.2956	<.0001	0.017
GP21	0.0658	0.112	0.557	1.068	-0.2736	0.0758	0.0003	0.761
GP22	0.9243	0.1569	<.0001	2.52	0.9301	0.0957	<.0001	2.535
GP23	0.623	0.1271	<.0001	1.864	-0.0731	0.0741	0.3235	0.929
GP24	1.2841	0.1732	<.0001	3.611	1.2623	0.1152	<.0001	3.533
GP3	-0.9371	0.1747	<.0001	0.392	-1.046	0.1119	<.0001	0.351

GP4	0.875	0.1408	<.0001	2.399	1.7972	0.1359	<.0001	6.033
GP5	-2.2468	0.2456	<.0001	0.106	-3.8454	0.2872	<.0001	0.021
GP6	0.5419	0.1238	<.0001	1.719	1.7009	0.1266	<.0001	5.479
GP7	-1.4483	0.1753	<.0001	0.235	-1.5589	0.1212	<.0001	0.21
GP8	-0.6294	0.1252	<.0001	0.533	-0.7117	0.0863	<.0001	0.491
GP9	-0.2203	0.1136	0.0524	0.802	0.1509	0.0759	0.0469	1.163
IGP24	0.0489	0.1119	0.6619	1.05	-0.3725	0.0807	<.0001	0.689
IGP25	1.4787	0.1832	<.0001	4.387	1.3999	0.1185	<.0001	4.055
IGP26	-0.2534	0.1177	0.0313	0.776	-0.9478	0.0993	<.0001	0.388
IGP27	0.9513	0.1516	<.0001	2.589	0.4353	0.0819	<.0001	1.545
IGP28	0.0711	0.1115	0.5235	1.074	0.044	0.0747	0.5559	1.045
IGP29	-0.3228	0.225	0.1514	0.724	-0.3402	0.1594	0.0328	0.712
IGP30	0.8895	0.1421	<.0001	2.434	0.7015	0.0901	<.0001	2.017
IGP31	0.4762	0.1317	0.0003	1.61	1.11	0.1044	<.0001	3.034
IGP32	1.6849	0.2095	<.0001	5.392	2.1653	0.1707	<.0001	8.717
IGP33	-1.3586	0.1752	<.0001	0.257	-1.5596	0.1284	<.0001	0.21
IGP34	-0.9769	0.1601	<.0001	0.376	-0.6395	0.0896	<.0001	0.528
IGP35	-0.8802	0.1629	<.0001	0.415	-0.7417	0.0988	<.0001	0.476
IGP36	0.9371	0.1759	<.0001	2.553	1.8223	0.1389	<.0001	6.186
IGP37	0.5406	0.168	0.0013	1.717	1.3919	0.1233	<.0001	4.022
IGP38	0.4646	0.1353	0.0006	1.591	1.2758	0.1129	<.0001	3.582
IGP39	0.3247	0.1161	0.0052	1.384	1.2731	0.1123	<.0001	3.572
IGP40	0.3265	0.1157	0.0048	1.386	1.1803	0.1049	<.0001	3.255
IGP41	0.244	0.1173	0.0374	1.276	0.5881	0.0867	<.0001	1.801
IGP42	0.4331	0.121	0.0003	1.542	1.3419	0.111	<.0001	3.826

IGP43	0.9867	0.1469	<.0001	2.682	1.8653	0.1391	<.0001	6.458
IGP44	-2.278	0.2492	<.0001	0.102	-4.0163	0.3012	<.0001	0.018
IGP45	0.5838	0.1254	<.0001	1.793	1.7349	0.128	<.0001	5.668
IGP46	-1.4599	0.1768	<.0001	0.232	-1.7004	0.1288	<.0001	0.183
IGP47	-0.6734	0.1273	<.0001	0.51	-1.3015	0.1086	<.0001	0.272
IGP48	-0.2482	0.1145	0.0302	0.78	-0.1088	0.0743	0.1431	0.897
IGP49	-0.6531	0.1309	<.0001	0.52	-0.4102	0.0782	<.0001	0.663
IGP50	-0.7907	0.1373	<.0001	0.454	-0.1414	0.0751	0.0597	0.868
IGP51	-0.3014	0.1146	0.0086	0.74	-0.6439	0.0824	<.0001	0.525
IGP52	-1.4673	0.1821	<.0001	0.231	-2.9846	0.2061	<.0001	0.051
IGP53	-0.3188	0.1165	0.0062	0.727	-1.2466	0.1063	<.0001	0.287
IGP54	-1.2078	0.1605	<.0001	0.299	-1.8939	0.1394	<.0001	0.15
IGP55	1.0325	0.1502	<.0001	2.808	2.1105	0.1518	<.0001	8.252
IGP56	-1.3333	0.1671	<.0001	0.264	-1.6833	0.1299	<.0001	0.186
IGP57	-0.4739	0.1223	0.0001	0.623	-1.3899	0.1127	<.0001	0.249
IGP58	0.536	0.1307	<.0001	1.709	0.5847	0.0903	<.0001	1.794
IGP59	-0.0613	0.112	0.5843	0.941	-0.5994	0.0889	<.0001	0.549
IGP60	1.3958	0.184	<.0001	4.038	1.5153	0.1329	<.0001	4.551
IGP61	0.3865	0.12	0.0013	1.472	0.2703	0.0803	0.0008	1.31
IGP62	0.4554	0.1197	0.0001	1.577	-0.0682	0.0741	0.358	0.934
IGP63	0.2765	0.1144	0.0156	1.318	-0.1862	0.0762	0.0146	0.83
IGP64	0.544	0.1241	<.0001	1.723	0.1715	0.0761	0.0242	1.187
IGP65	0.9019	0.1435	<.0001	2.464	0.565	0.0904	<.0001	1.759
IGP66	-0.3296	0.1166	0.0047	0.719	0.2526	0.0765	0.001	1.287
IGP67	-0.3191	0.1158	0.0058	0.727	0.0237	0.0745	0.7509	1.024

IGP68	-0.3568	0.1179	0.0025	0.7	0.0109	0.0745	0.8836	1.011
IGP69	-1.0485	0.155	<.0001	0.35	-0.7208	0.0871	<.0001	0.486
IGP70	-0.356	0.1171	0.0024	0.7	0.2216	0.0759	0.0035	1.248
IGP71	-0.3573	0.1173	0.0023	0.7	0.2138	0.076	0.0049	1.238
IGP72	0.4142	0.119	0.0005	1.513	-0.1159	0.0748	0.1213	0.891
IGP73	-1.4643	0.1818	<.0001	0.231	-2.9697	0.2054	<.0001	0.051
IGP74	-1.0613	0.1545	<.0001	0.346	-0.7288	0.0879	<.0001	0.483
IGP75	-1.0653	0.1552	<.0001	0.345	-0.7376	0.0873	<.0001	0.478
IGP76	1.2463	0.1656	<.0001	3.477	1.0149	0.0976	<.0001	2.759
IGP77	-1.3632	0.1786	<.0001	0.256	-2.0073	0.1567	<.0001	0.134
